# Dr. Jenner's Letter on the Vaccine Inoculation

**Published:** 1800-02

**Authors:** E. Jenner

**Affiliations:** Berkeley


### *To the Editors of the Medical and Phyfical Journal.*

Gentlemen,

In your Journal for the prefent month, I obferve an extract from a paper fent you by Dr. Pearfon, on the appearance of puftules

---

puftules refembling the fmall-pox in the vaccine inoculation. When I have had an opportunity of perufing this paper, I may probably requeft the favour of you to give room to fome comments upon it; in the mean time, it may be proper to remark, that from the commencement of my inoculation with the vaccine virus to the prefent day, no puftules, fimilar to the variolous, have in any one inftance appeared. I have feen rafhes, and fometimes (though very rarely) I have obferved a few fcattered pimples about the body; indeed, it has often appeared fingular to me, on reflecting how frequently local cuticular inflammation and irritation (whether arifing fpontaneoufly, or from the application of acid fubftances) occafion fuch appearances, that they fhould have occured fo feldom.

Time will develope the myftery before us; at prefent, I very much fufpect, that where *variolous puftules* have appeared, *variolous matter* has occafioned them. The many obfcure ways by which it may effect the fyftem, muft be too obvious to require explanation.

I remain, GENTLEMEN,
Your obedient humble fervant,
E. JENNER.

*Berkeley, Jan.* 13, 1800.

